# The visual basis of reading and reading difficulties

**DOI:** 10.3389/fnins.2022.1004027

**Published:** 2022-11-23

**Authors:** John Stein

**Affiliations:** Department of Physiology, Anatomy and Genetics, University of Oxford, Oxford, United Kingdom

**Keywords:** dyslexia, phonology, temporal processing, magnocellular, visual, color filters, omega 3, auditory

## Abstract

Most of our knowledge about the neural networks mediating reading has derived from studies of developmental dyslexia (DD). For much of the 20th C. this was diagnosed on the basis of finding a discrepancy between children’s unexpectedly low reading and spelling scores compared with their normal or high oral and non-verbal reasoning ability. This discrepancy criterion has now been replaced by the claim that the main feature of dyslexia is a phonological deficit, and it is now argued that we should test for this to identify dyslexia. However, grasping the phonological principle is essential for all learning to read; so every poor reader will show a phonological deficit. The phonological theory does not explain why dyslexic people, in particular, fail; so this phonological criterion makes it impossible to distinguish DD from any of the many other causes of reading failure. Currently therefore, there is no agreement about precisely how we should identify it. Yet, if we understood the specific neural pathways that underlie failure to acquire phonological skills specifically in people with dyslexia, we should be able to develop reliable means of identifying it. An important, though not the only, cause in people with dyslexia is impaired development of the brain’s rapid visual temporal processing systems; these are required for sequencing the order of the letters in a word accurately. Such temporal, “transient,” processing is carried out primarily by a distinct set of “magnocellular” (M-) neurones in the visual system; and the development of these has been found to be impaired in many people with dyslexia. Likewise, auditory sequencing of the sounds in a word is mediated by the auditory temporal processing system whose development is impaired in many dyslexics. Together these two deficits can therefore explain their problems with acquiring the phonological principle. Assessing poor readers’ visual and auditory temporal processing skills should enable dyslexia to be reliably distinguished from other causes of reading failure and this will suggest principled ways of helping these children to learn to read, such as sensory training, yellow or blue filters or omega 3 fatty acid supplements. This will enable us to diagnose DD with confidence, and thus to develop educational plans targeted to exploit each individual child’s strengths and compensate for his weaknesses.

## Introduction

Human language evolved around 100,000 years ago, whereas writing was invented much later, only around 6,000 years ago. Also until the last 100 years or so, only a small proportion of humanity ever had to learn to read, and many of these were priests who were celibate, so they did not pass their genes onward. In consequence, whereas language is innate and most children instinctively copy their mothers and thus learn to talk, children usually have to be explicitly taught how to read. But in the UK and USA around 1 in 5 fail to do so, because our education systems are underfunded and reading is the most complicated cognitive skill that everybody is now expected to acquire. Here we will briefly review mainly the visual mechanisms underlying reading and their implications with regard to helping those who are finding the process difficult. But let it be emphasized at the outset reading is multifactorial; in particular not only accurate visual, but also auditory, processing is required.

Until recently most of our knowledge about the neurology of reading derived from observations on people who had failed to do so. The majority of those who fail, do so through low general intelligence and/or for social reasons: a toxic mixture of poor teaching, truancy, lack of parental support in impoverished and disadvantaged families. However, unfortunately the neural mechanisms underlying their failure are not studied much, and most studies are in children with normal or high intelligence and good family support, but who, despite these advantages, for some reason fail to learn to read fluently. These are the children who are said to have “developmental” dyslexia.

The word dyslexia, was derived by Berlin (1884) from the Greek “dys” meaning disordered, and “lexis” meaning words. He applied this word to the rare stroke patients he saw in whom cortical damage had selectively deprived them of the ability to read, but had not affected their speech, comprehension, nor their non-verbal reasoning powers. We now call this condition “acquired dyslexia.” Dejerine even found patients who could no longer read, but could still write normally—“dyslexia without dysgraphia.” Berlin thought the problem was mainly visual. So he also called it “word blindness.” A few years later James Kirk and J. Pringle Morgan postulated that there might be a developmental analog of the brain damage condition, in which, for some reason, children fail to properly develop the brain circuits which mediate reading—“developmental dyslexia (DD).” His now famous patient, 14 year old Percy, was normally intelligent in oral conversation. But despite 9 years of schooling he hadn’t even managed to learn to read or spell his own name properly yet (Morgan, 1896).

Morgan also noted that there were many persons in Percy’s family with the same sorts of problem. So he speculated that the condition was “congenital.” In the UK, Morgan, Kerr and Hinshelwood, and in the USA, Samuel Orton, all thought that it was a subtle visual processing problem, and continued to call it word blindness as well. But the term, developmental dyslexia (DD), soon replaced it because it became clear that sometimes this was not primarily a visual problem. It was assumed that DD was a genetically based failure to properly develop the correct brain connections for reading. And the early pioneers in the subject were confident that future research would uncover what these were. Meantime, the condition could be identified by demonstrating a big difference between a child’s normal or high oral and non-verbal intelligence, yet markedly backward reading and spelling, together with a family history of similar problems, suggesting a genetic basis. This “discrepancy” criterion, together with the strong family history, served to identify and diagnose DD successfully for nearly 100 years.

## The phonological theory

However, in the second half of the twentieth century the neurological approach to children’s reading problems was gradually replaced by a linguistic/psychological one, because reading problems were, by then, mainly encountered by teachers and educational psychologists rather than neurologists. This process was especially influenced by the revolutionary ideas of Noam Chomsky. In particular, he showed how “recursion”—the repeated application of the same rule to its own output—could generate from a small number of speech sounds, “phonemes,” a potentially infinite number of different words (Chomsky, 1957). This “phonological principle” underlies all language and writing. Accordingly, it was soon argued that DD was due to failure to acquire this phonological principle, without any visual processing problems (Liberman et al., 1971).

However, this theory does not attempt to explain *why* some children fail to grasp the phonological principle. Indeed it is almost a tautology, since it merely repeats, using different words, that the children have failed to learn to read, since the fundamental skill underlying reading is to learn to translate letters into the sounds (phones) they represent. Any child who cannot read has failed to acquire the phonological principle; hence all such children have a phonological deficit. But importantly there are numerous reasons, other than DD, why a child may fail to grasp this principle and fail to learn to read. These other causes are usually an interacting complex of social, rather than neurological, factors, such as poverty and deprivation, poor teaching, truancy, home chaos, low expectations and lack of family support. However, all the causes, including DD, manifest as failure to learn and absorb the phonological principle. Accordingly, almost all poor readers will fail phonological awareness tests, so low scores on such tests do not distinguish DD from any of the other causes. Hence, if we attempt to use these tests alone to identify DD, all poor readers will be diagnosed as dyslexic, and the diagnosis would become pointless because it would not point to any specific cause or to any treatment that might help them (Elliott, 2020).

## The current situation

Indeed, this is what seems to be happening nowadays to a worrying extent. Almost everyone whose reading or other educational achievements are behind that expected for their chronological age may be called “dyslexic.” Currently, practitioners are dissuaded from looking for discrepancies between reading and oral language competence to diagnose it, but instead they are instructed to emphasize phonological tests. As we have seen, since almost all poor readers fail these tests, they don’t distinguish DD from other causes of reading failure, hence they cannot be used to identify it.

In reality, however, assessors actually do use discrepancy criteria, but covertly—for example by requiring a child to have at least a “normal” IQ for their age, yet recording more than 1.49 sds below average in their reading, phonological or temporal processing scores, in order to be classified as dyslexic. However, full scale IQ tests are highly unsuitable for measuring oral and non-verbal ability, since they involve a lot of reading (Thomson, 1982).

Furthermore, and in practice more importantly, measuring IQ and administering a variety of other psychometric tests takes up a great deal of time and expense, which has meant that only the relatively wealthy middle classes can afford to have their children tested privately. Many UK Education Authorities have now abandoned testing for DD at State expense, or even using the term at all. Now also practitioners look for many different possible discrepancies. The consequence is that the diagnosis of DD now covers a wide variety of learning disabilities and doesn’t accord at all with the “classical” definition. The combination of the cost and the unclear criteria for identifying dyslexia specifically, has made the diagnosis inconsistent between practitioners and overall a muddle, and this is what has prompted some people to suggest that we abandon the concept altogether (Elliott and Grigorenko, 2014).

## Return to discrepancy criterion?

This unhappy situation has arisen as a result of replacing the classical oral intelligence/reading discrepancy with some form of a phonological awareness/chronological age discrepancy definition, because the latter does not distinguish it from any of the other possible causes of reading failure. The solution should surely be to return to the classical reading/oral language discrepancy definition of DD which had served us well for so long, whilst awaiting the development of more objective biomarkers derived from our growing understanding of the physiology of what causes some children to experience such difficulties learning to read, despite having normal oral and non-verbal intelligence. Growth of such understanding was confidently expected by the likes of Morgan, Orton and Hinshelwood to explain how a hereditary disposition can cause learning to read to fail to progress normally in some children; and recent advances in the genetic approach suggest that such biomarkers will indeed be forthcoming in the near future (Mascheretti et al., 2018).

## Genetic background

One of the few things that nobody disputes is that DD is strongly hereditary. Twin and family studies all agree that its heritability is around 60% (Olson, 2006). But only for rare single gene disorders is one gene alone, ever wholly responsible for a condition. None of the dozen or so gene variants, nor any of the more than 60 single nucleotide polymorphisms (SNPs) that have been shown to be associated with dyslexia, individually explain more than a tiny proportion of its heritability. Consequently so far, studying the genetic basis of reading difficulties has not added much to our understanding of the neural basis of reading. Nevertheless one of these genes, ROBO1, which helps to guide axons to their correct destinations during brain development, has been found to be associated specifically with visual motion sensitivity (a putative visual temporal processing marker) in dyslexics (Mascheretti et al., 2020). This is perhaps a first step in unraveling how gene variants might endow impaired visual processing to people with dyslexia.

## Visual precedes phonological analysis

The great emphasis now placed on children acquiring the phonological principle is especially perverse in view of the fact that it was confirmed more than half a century ago what had been assumed for much longer, that visual processing is the actual starting point for learning to read (Morais et al., 1979). When children, or indeed illiterate adults, are first confronted with a written word they don’t automatically see it as a series of letters, but as a single object like a mouse. When you see a mouse, you don’t dissect it into a sequence of whiskers, nose, ears, head, body, tail, instead you see it as a single mouse. So the first thing somebody has to do to learn to read, is to learn to visually dissect words into their sequences of letters. Morais et al. (1979) showed that it is not until a child, or even an illiterate adult, has learnt that a written word consists of a sequence of letters, that they can begin to grasp that its spoken form consists of a series of more elementary sounds, “phonemes,” which the letters in its written form stand for. So learning to sequence letters visually primes learning to sequence its sounds aurally. Thus visual sequencing always precedes phonological analysis. Often therefore, deficient visual processing is the cause of a dyslexic child’s reading problems, but in others deficient auditory processing can be the main cause.

## Timing and sequencing

Sequencing letters visually requires *timing* when and where a person sees the first letter, then the next and so on. This timing and rough localization depends on a subsystem of vision—the magnocellular system (from the Latin magnus meaning large) (Nassi and Callaway, 2009). 10% of the ganglion cells in the retina are these magnocells (M- cells) which are much larger than the rest, having receptive fields up to 50x larger than the much more numerous and smaller parvocells (from L. parvus meaning small). Being larger, magnocells have thicker axons and therefore conduct impulses into the brain much more rapidly than the P-cells; the M- cell volley normally reaches the visual cortex c. 10 ms earlier than the P-cells’ (Maunsell et al., 1999). The M- cells’ large receptive fields mean that they cannot signal fine detail, such as that which distinguishes one letter from another. Thus M- cells could not distinguish between the “d” and the “g” in the word “dog,” for instance. But by directing the focus of attention to the right spot, they instruct P- cells to make that distinction (Vidyasagar and Pammer, 2010). Thus M-cells can signal when the eyes and focus of attention first alight on the “d” of “dog,” then the “o,” then the “g”; and clocking these movements enables representation in short term memory of the order in which the letters were seen. Both acquired and developmental dyslexics are conspicuously bad at correctly sequencing letters correctly.

The main reason for describing magnocellular and parvocellular retinal ganglion cells here, is because there is now overwhelming evidence that many, if not all, developmental dyslexics show impaired development of their visual magnocellular systems (Gori et al., 2016; Stein, 2019). This expresses itself not only in the retina, but in all the brain regions to which the magnocellular system provides significant input. Because magnocellular neurones provide rapid signaling of when and where visual events occur in the external world, their main function is to detect movement, for the visual guidance of attention and of the movements of the eyes and limbs. These functions are mediated by their dominant (90%) input to the “dorsal attentional system.” This runs from the primary visual cortex forward via V5 (also known as MT) and the posterior parietal cortex (PPC) to the dorsolateral prefrontal cortex (dPFC) (Laycock et al., 2008). Impaired visuomotor function has been demonstrated in all these areas in dyslexics. Space forbids presenting all this evidence here, but some of the most persuasive will now be discussed.

## Selective stimulation of magnocells

Much of this evidence showing that many children with dyslexia have impaired development of their visual magnocellular timing systems, depends on our ability to selectively stimulate these cells, rather than other retinal cells. This is possible because M- cells are most sensitive to low contrast, coarse, brief flashes of yellow light, operationalized as low contrast, low spatial and high temporal frequency stimuli, whereas parvocells are most sensitive to the converse: high contrast, high spatial but low temporal frequency stimuli. Therefore, the most convincing experiments that demonstrate specific abnormalities of the magnocellular system in dyslexics are those that employ low contrast, low spatial and high temporal frequency stimuli, and which compare these with their responses to high contrast, high spatial and low temporal frequency stimuli, to which parvo- cells respond better. These experiments have demonstrated that dyslexics’ responses are significantly reduced to the former, but normal or even increased to the latter. This comparison also rules out the possibility that the people with dyslexia may be simply worse at all visual tests due to lack of attention or simply not bothering.

## Retina

We will now consider experiments at the different levels of the visual system that demonstrate that in many dyslexics M-cell function is impaired, whereas P- cell function is normal or even enhanced. Lovegrove was the person who really initiated the magnocellular theory of dyslexia. He was the first to demonstrate that dyslexics have reduced sensitivity to gratings of low contrast and low spatial frequency flickered at high temporal frequencies (Lovegrove et al., 1980). Since M- ganglion cells respond best to stimuli with these properties, he suggested that the visual “transient” system in dyslexics was defective; this was the term used in 1980 for the visual magnocellular system. There have been innumerable studies that have confirmed his hypothesis as we shall see. Lovegrove also showed that in some circumstances people with dyslexia were actually more sensitive than ordinary readers to high contrast, high spatial frequency, low temporal frequency stimuli. This suggests that the parvocellular systems in dyslexia may actually be more sensitive, than those of ordinary readers (Lovegrove et al., 1982), a possibility that we will consider in greater detail later on.

The simplest low spatial and high temporal frequency stimulus is just a flickering light, and there have been numerous studies showing that in many dyslexics the frequency at which a flickering light ceases to appear to flicker, but becomes continuous (the so-called critical flicker fusion frequency- CFF), is significantly lower (Brannan and Williams, 1988; Johnston et al., 2017). Indeed the magnitude of this reduction predicts the degree of their reading failure (Talcott et al., 1998). This suggests that this magnocellular deficit may indirectly cause the reading problem, though of course it does not prove this. But this reduction in the CFF is too small and variable to use diagnostically on an individual basis.

Magnocellular retinal ganglion cells respond “non-linearly”; they are ON/OFF cells, discharging as well to a light switching off as to switching it on, which means that they fire most at the 2nd harmonic of the frequency of a light whose intensity is varied sinusoidally. Thus M- cells give a larger response at the 2nd harmonic, whereas linear P cells do so mainly at the fundamental frequency. Hence typical readers give a larger cortical response at the second harmonic, but dyslexics do so at the fundamental frequency, and this phenomenon has been exploited by comparing the fundamental and 2nd harmonic peaks in steady state visual evoked potentials recorded from dyslexics as a simple test of their magnocellular sensitivity (Stein, 2021).

This non-linear property also explains why black and white stripes, switched on and off 10 times per second or faster, perceptually appear to be twice as fine as they actually are. This is the “spatial frequency doubling illusion” (Rosli et al., 2009). Dyslexics have been shown to need a higher contrast to see these gratings at all (Pammer and Wheatley, 2001) confirming that their M- cells are less sensitive. Again, the increase in contrast they require to see the gratings predicts their degree of reading failure.

## Lateral geniculate nucleus

The axons of retinal magnocellular neurons project to the magnocellular layers (1 & 2) of the lateral geniculate nucleus (LGN) in the thalamus. 30 years ago, Livingstone et al. (1991) reported the results of a histological examination of the brain of some 70 year old dyslexics, who had first been seen by Samuel Orton in the 1920s. His patients had bequeathed their brains to the Orton brain bank, now in Harvard. Livingstone and Galaburda found that the LGN M-cells in these brains were significantly smaller compared with those in a control brain. Also they were much more disorganized, encroaching into the koniocellular area which normally clearly separates layers 2 and 3 from each other; in other words they had migrated too far during development. In the same brains Galaburda et al. (1985) had already shown similar excessive migration in the cerebral cortex of the dyslexic brains that had caused an unusually large number of “ectopias” (brain warts) to form on the cortical surface, particularly involving left hemisphere language areas.

Nowadays, higher strength 7 Tesla magnets have increased the spatial resolution of morphological MRI imaging down to less than a millimeter, which is sufficient to resolve the separate layers of the LGN, in life. Accordingly Giraldo-Chica et al. (2015) have now confirmed Livingstone’s results in 15 awake volunteer dyslexics. The magnocellular layers of their LGNs were significantly thinner than in typically developing readers, particularly on the left, language, side.

## Visual cortical areas

As far as the primary visual (striate) cortex, area V1, situated at the back of the occipital lobe, M- and P- inputs are anatomically separated. But in V1 they interact extensively. Accordingly, the M- “stream” cannot be said to be “pure” M- thereafter (Skottun and Skoyles, 2007). Nevertheless, as well as supplying 90% of the input to the dorsal attentional pathway mentioned earlier, M- cells actually also provide 50% of that to the ventral occipital form and pattern analyzing system, the other main visual pathway passing forward to the infero- temporal cortex. The function of this M-input to the ventral pathway is believed to be to draw attention to what part of visual space needs to be analyzed in detail by the P- system (Vidyasagar and Pammer, 2010).

## The visual motion area- V5/MT

Thus the “visual motion area” at the front of the occipital lobe (V5, also known as MT) receives most of its input from retinal magnocells; and most of the neurons there are directionally selective, meaning that they have become specialized for detecting visual motion. In fact, they, like all the cells in the dorsal stream, are genetically related, as indeed are all the “transient” systems in the brain, because they all express similar surface “signature” molecules by which they recognize each other to make preferential connections. Thus M- like cells can be identified anywhere using antibodies specific for that lineage, such as CAT 301 (Hockfield and McKay, 1983); and they are found all over the brain.

The sensitivity of an individual’s visual motion system can be tested psychophysically by measuring how many otherwise randomly moving dots on a screen have to move in the same direction, “coherently,” in order for an observer to determine in which direction they’re going (Britten and Newsome, 1992). The smaller the proportion required, the greater his motion sensitivity, and numerous studies have confirmed that dyslexics, as a group, have lower sensitivity than ordinary readers to these “random dot kinetograms” (RDKs). Furthermore the lower an individual’s sensitivity, the worse his reading is (Cornelissen et al., 1995; Franceschini et al., 2012). However, again, individual responses are so variable that this test cannot be used for diagnosis in individuals.

## Visual event related potentials

Sensitivity to visual motion can also be indexed by recording Visual event related potentials (VERPs). This was first carried out by Livingstone and Galaburda in 1991 (Livingstone et al., 1991). They measured visual evoked potentials in response to a moving checker board stimulus in 5 dyslexics and found that their latency was longer and amplitude smaller compared with 7 good readers. This result has been confirmed many times, in much larger samples and with more advanced technology (Klistorner et al., 1997; Schulte-Körne and Bruder, 2010; Jednoróg et al., 2011; Stein, 2021) and the result is no longer seriously doubted, although whether the deficit is due to undersampling due to smaller and sparser retinal magnocells, longer integration times or increased local noise, is still debated (Manning et al., 2019).

## Functional magnetic resonance imaging

Advances in Functional magnetic resonance imaging (fMRI) have enabled the VERP results to be confirmed with much better spatial resolution using this technology. Thus Eden (Eden et al., 1996) was the first to show reduced activation of V5/MT in dyslexics viewing a moving pattern, compared with controls, an observation that has since been replicated by several other labs. Again, the size of the reduction predicts the subjects’ degree of reading difficulty (Demb et al., 1998), but this expensive technique is unlikely to become useful for individual diagnosis.

## Eye movement control

Having considered the evidence for impaired magnocellular development in DD at each level of the visual system, we will now discuss how this affects the cortical systems in which the M- system plays an important part in their control. Stable fixation on letters or words being inspected is obviously important for successful reading and this stability depends crucially upon detecting any motion caused by unwanted eye movements which may cause letters to appear to move around. This motion signal is fed back to the ocular motor control system, which negates it by directing the eyes back on to the target. A weak M system therefore leads to less stable visual fixation, which in turn leads to words and letters appearing to move around. This is a problem which many dyslexics experience (Fowler and Stein, 1979; Singleton and Trotter, 2005; Harries et al., 2015). Hence M- impairment impacts on dyslexics’ eye movement control very significantly (Eden et al., 1994; Kirkby et al., 2008; Jainta and Kapoula, 2011), and the stability of the fixation of their eyes on letters is significantly reduced (Raymond et al., 1988; Fischer and Hartnegg, 2000).

## Vergence

In addition when reading, the two eyes need to converge precisely to focus on letters 30 cms away, and the M- system is crucially involved in the first stage of controlling these convergence eye movements (Mowforth et al., 1981). But the vergence eye movement control system is highly vulnerable to drugs and disease, as we know to our cost if we consume too much alcohol; our eyes cease converging properly and things can seem to go double (diplopia). We and many others have shown that many dyslexics have this kind of unstable vergence control (Fowler and Stein, 1983; Liversedge et al., 2006; Bucci et al., 2007), hence they have a pronounced tendency to experience diplopia when attempting to read.

Furthermore, in individuals the degree of reduction in their visual motion sensitivity predicts the extent of their eye instability problems (Ray et al., 2005). Indeed, motion sensitivity, i.e., M-cell sensitivity, predicts orthographic reading skill, not just in dyslexics, but in everyone (Witton et al., 1998). Thus, because all eye movements depend on M- control, M- insensitivity leads to impaired control of all kinds of eye movement. For instance, when the eyes track a moving target (in “smooth pursuit”), dyslexics tend to fall progressively behind it; so they have to make periodic saccades in order to catch up. Thus such “saccadic intrusions” are much commoner in dyslexics (Adler Grinberg and Stark, 1978; Eden et al., 1994).

In people with dyslexia, the accuracy of saccades is impaired whatever the target, not just when they are reading (Biscaldi et al., 2000). Thus much of the evidence for their impaired eye movement control is derived from recording their responses to targets other than text, i.e., not involving reading at all. This provides yet more evidence suggesting that their poor eye control is a cause of their impaired reading, rather than being just a result of it.

Nevertheless many people still believe that all the eye movement abnormalities found in dyslexics are the result of their difficulties with decoding, rather than their cause (Rayner, 1998). There is no doubt that increased numbers of regressive saccades (returning to words not successfully decoded) and prolonged fixations (due to their longer decoding time) may be partly caused by their decoding problems. But this does not explain why their fixations are so unstable, nor why the eyes diverge inappropriately and cause diplopia, nor why these problems occur when inspecting any visual sequences, not just text.

## Visual attention

Because the visual magnocellular system guides attention as well as eye movements, M deficiency leads to slower, less accurate deployment of visual attention (Vidyasagar, 2005). “Serial visual search” is when each in an array of similar targets has to be inspected, one after the other, in order to detect a particular one, whereas “parallel search” is when a feature in one of the objects is so distinctive it just “pops out.” Dyslexics are as good or better at parallel search, but much slower at serial search (Vidyasagar and Pammer, 1999; Facoetti et al., 2000; Iles et al., 2000), and this explains why they are slower and less accurate at any kind of visual sequencing, not just of letters.

Text is an extremely “crowded” visual stimulus. Amidst the distracting surrounding text, the M- system normally directs our attention accurately onto the particular word we are trying to comprehend, but the closer letters and words are to each other, the more difficult it is to concentrate on just this one word. This crowding interferes greatly with the ability to pick it out and accurately read it. This problem is much more pronounced in dyslexics than in good readers. So using more widely spaced print often helps them considerably (Cornelissen et al., 1991; Martelli et al., 2009).

As we have seen the essence of learning to read is learning to associate the shape of a letter with the sound it represents and that the letters translate into those particular sounds. So visual attention must be properly cued to those sounds. But this visual/auditory crossmodal cueing of attention is greatly impaired in those with dyslexia (Gabrieli and Norton, 2012; Harrar et al., 2014).

In order to argue against the possibility that a visual processing deficit may contribute to dyslexia, it is often suggested that people with dyslexia are just bad at all tests, due to a general lack of concentration and motivation; so it is argued that there may be nothing specific about their impaired M- function, but they just don’t try hard enough. However, if this were so, they would be equally bad at detecting stimuli designed to stimulate P cells selectively. As we have seen, many studies have compared dyslexics’ M- sensitivity to their P- sensitivity to stimuli such as static visual forms, and found it normal. Indeed, as mentioned earlier, they may actually have higher contrast sensitivity for functions mediated by the parvo system, e.g., at high spatial and low temporal frequencies (Lovegrove et al., 1982) and they have better color discrimination in the visual periphery (Dautrich, 1993), possibly because they have more P-cells in the peripheral retina.

## The reading connectome

Until recently, the neurological approach to brain function, cognitive skills and disease rested on the classical idea of “localization of function.” For each cognitive function, it was argued, there would be a single specific cortical area that is mainly responsible. But as we learn more about how brain structures interact, it has become clear that whilst the primary sensory and motor areas are indeed functionally localized, in the rest of the cortical “association areas,” it is more useful to consider how they connect with each other, their “connectomes,” than to concentrate on a single area (Dick et al., 2013). Recent advances in functional MRI, in particular diffusion tensor imaging (DTI), allows the functional connectivity between areas to be traced and also to investigate how these change not only over the long term during development (Feng et al., 2022), but also over shorter terms to mediate the acquisition of new cognitive skills, such as reading.

To simplify drastically, the cerebral cortex can be seen as a network of nodes connected to each other by either long or short interconnections, and although these are initially set up by the genes you inherit, they develop continuously throughout life, but particularly in childhood, according to your environment, nutrition, education, experiences, actions, emotions and memories. Those that contribute to successful processing survive; those that do not are eliminated. For reading, the optimal end result is a “small world” network, richly connected to neighboring, but sparsely connected to distant nodes (Bullmore and Sporns, 2012). Thus in a recent UK study, reading and maths networks were compared in a large number of children covering the full range of abilities in these two domains. There was no evidence that the poorer readers had missing connections or nodes compared with the good readers, but the strength of some crucial connections were much weaker in the poorer readers compared with the better ones (Bathelt et al., 2017).

Dyslexia seems to be equally common in the very different Chinese character script (Peng et al., 2017). Given the very visual nature of the characters it was not surprising that network analysis showed that fluent adult Chinese readers develop significantly stronger visually based connections than do children who have not yet learnt to read Chinese fluently (Zhou et al., 2021).

## Cause or effect? Reading age matches

One problem with many of the results that we have discussed so far, is that reduced M cell sensitivity could conceivably be a result rather than a cause of failing to learn to read, because the children will have had less practice at the required visual skills (Goswami, 2015), and therefore might have failed to develop them because of this lack of exposure. One way of avoiding this problem is to compare dyslexics’ M sensitivity with that of younger, typically developing children, matched for reading age with the dyslexics. This is known as a “reading age match” design. Under these conditions, these younger children will have had no greater experience of reading than the older dyslexics have had, and therefore their M- sensitivity should be no better than that of the dyslexics if visual experience were the crucial factor. However, as we have seen, many studies have shown clearly that younger readers who have had the same amount of reading experience as people with dyslexia, have already developed much better visual M- function (Gori et al., 2016).

Nevertheless it is clear that impaired visual M- function alone is neither a sufficient, nor a necessary cause of dyslexia, and therefore cannot be advanced as its sole cause. But it probably makes an important contribution in most dyslexic people. As we shall see, however, auditory and probably other neural temporal processing problems may also be relevant.

## Cohort studies

Another powerful way to demonstrate that timing and sequencing deficits may be a cause of children’s reading problems, rather than merely their consequence, is to look for M- deficits in children before they even begin to learn to read. The best way to do this is to select children at genetic risk of dyslexia on the basis that close relatives in the family have been diagnosed as dyslexic, so they have a much greater risk of becoming dyslexic themselves. Better still, make this a “cohort” study, where the children at genetic risk of dyslexia are studied both before they begin to learn to read and then again at intervals after they have or have not managed to do so. Two large studies of this sort have been carried out, one in Finland (Hamalainen et al., 2008) and the other in Holland (van der Leij, 2013). They have both shown that neurological differences manifest themselves in infants, and even in newborn babies, in those who are going to go on to become dyslexic later on. In the Dutch study of children at family risk, habituation to a simple visual, non-alphabetic, stimulus measured by ERPs at the age of 5, before learning to read had commenced, predicted whether the child would be identified as dyslexic by the age of 8 (Regtvoort et al., 2006). Thus these cohort studies have established that impairments in temporal processing precede a child’s failing to learn to read and therefore strongly support the hypothesis that they contribute causally to any subsequent failure.

## Intervention studies

However, the most convincing way of demonstrating that impaired visual magnocellular performance causes visual difficulties with reading is to show that improving it by training, or in other ways, helps children to overcome those difficulties and to make significant progress with their learning to read. Ideally, we would show this by means of randomized control trials (RCTs). Unfortunately given the opposition to the whole concept (leading to lack of funding) only a few of these have been carried out. Even though all have come to positive conclusions, none of them have been large enough to overcome the opposition. Space forbids much detail here, but we can consider some of this evidence.

Lawton has spent a lifetime developing a system based on the known properties of the M-system for training poor readers with an initially weak M-system (Lawton, 2016). She uses a low contrast, low spatial frequency, moving grating presented against a static background grating of higher contrast and spatial frequency. Subjects have to report the direction of motion whilst the program iteratively reduces the contrast of the moving grating as their threshold for correct responses improves. This training procedure greatly helps the children to improve their M- sensitivity. Thus she can simultaneously measure the children’s current motion sensitivity, how this improves with her training and whether this enhances their reading progress. And this has shown clearly that this training helps the majority of dyslexic children to improve their M- sensitivity and thereby make considerable progress with their reading, thus offering powerful evidence that their improved M- function was what enabled their improved reading (Lawton and Stein, 2022).

## Action video games

Another way of training M- function, not involving reading, that has been used by many labs is to ask the children to play action videogames. These incorporate the active tracking of moving targets with the eyes and limbs (Bavalier and Davidson, 2013), obviously engaging the M- system. These games have proved to be extremely effective in improving children’s M- function. Accordingly, their ability to focus visual attention and concentrate on the task in hand increases. Indeed, not only is the direction of visual attention improved, but also that of auditory attention (Mancarella et al., 2022) consequently these changes are followed by impressive improvements in their reading (Peters et al., 2019).

## Color filters

Perhaps the most controversial way of trying to improving magnocellular function is the use of color filters. These have mainly been used on the basis of other theories of why children can have visual reading problems. Here we will discuss their relevance to the magnocellular theory.

## Yellow filters

M-cells do not contribute to color vision, but they receive mainly from the retinal cone photoreceptors that are sensitive to Long (“red”) and Middle (“green”) wavelengths. Hence they are maximally stimulated by the combination of these two wavelengths, which is yellow light. This color is often called “unique yellow” because most observers deem it pure yellow, not tinged with red or green. It is also the dominant wavelength of day light at midday. Band pass filters passing mostly yellow light at 575 nM actually increase the amount of yellow light falling on the retina because with less light entering the eye overall, the pupils dilate. We have shown that viewing text through such filters helps some, but not all, children with visual problems when reading, to overcome them, hence to progress faster, sometimes dramatically. Since we showed that wearing these filters also improved the children’s visual motion sensitivity, fixation stability and vergence control we concluded that the yellow filters had improved the children’s M- function, hence that this was what enabled their reading progress (Ray et al., 2005).

## Blue filters

Another group of dyslexics seem to benefit from viewing text through the opposite color, “unique blue” filters (Clisby et al., 2000). These pass most light at c. 475 nanometers—Oxford blue. Unique blue filters probably work via the brain’s internal clock in the hypothalamic suprachiasmatic nucleus (SCN). This clock needs to be synchronized with sunrise which changes throughout the year. At sunrise the first rays of the sun in the morning are blue, and they are detected ganglion cells in the retina that have recently been discovered which respond especially to morning light, because they contain a blue sensitive pigment, melanopsin (Spitschan, 2019). They project to the hypothalamus and specifically activate the M- timing cells via a connection from the SCN to another “blue nucleus,” the locus coeruleus (coeruleus, Greek—blue). Blue light thereby increases general arousal and with it children’s ability to concentrate, and this is probably how it helps them to improve their reading.

Thus all these treatment interventions that target the magnocellular timing systems can help children with dyslexia to learn to read. Hence they add to the mounting evidence that impaired M- cell development throughout the CNS is an important contributor to dyslexic people’s reading problems.

## Criticisms, controversies and counter evidence

In summary, after the introduction of the visual transient/magnocellular hypothesis of dyslexia, the great majority of studies asking whether a visual magnocellular impairment can be detected, have found that indeed it can, in at least some dyslexics. In addition, visual attention, visual search and eye movement control are all agreed to be mainly mediated by the M-system. So the many experiments that have demonstrated deficiencies in these systems in dyslexics can be confidently interpreted as supporting the magnocellular hypothesis. Finally, as we have seen, there are now a large number of studies aimed at bolstering M- sensitivity either directly or indirectly, and most of these have demonstrated convincingly that any technique which improves M- cell function can help children to improve their reading.

With such strong evidence, one might well ask why this idea is so often dismissed as disproven, too highly controversial or simply ignored. The main reason is probably the overwhelming dominance of the phonological theory. But as we have seen this theory is more of a tautology, merely repeating that the children find reading difficult, rather than explaining why they fail. Yet many people mistakenly believe that it rules out any visual processing explanation for dyslexia. 50 years ago, however, the work of Morais (Morais et al., 1979) and a succession of later research, has clearly demonstrated the phonological deficit in dyslexics is likely to be often caused by their visual sequencing failures.

Nevertheless, a visual magnocellular weakness cannot be detected in every dyslexic; moreover some children with mildly impaired magnocellular function may still learn to read. Hence a visual M- deficit cannot be said to be the only cause of dyslexia (Skottun and Skoyles, 2004). Yet M- cell sensitivity predicts orthographic skill in both good and bad readers, whether or not they are classed as dyslexic (Talcott et al., 2002). Thus visual M- function appears to contribute significantly to everybody’s ability to acquire reading skills. But visual M- weakness seems to confer vulnerability to reading problems, but it is not their sole cause.

## Auditory temporal processing

Probably auditory temporal sequencing is also deficient in many people with dyslexia. After learning to sequence the letters in a word visually, the next stage in learning to read is to learn to translate this sequence into the sequence of sounds in the spoken form of the word. This requires the child to learn to time the order in which the sounds occur in the word. This requires precise auditory timing. The auditory timing system has transpired to be remarkably analogous to the visual timing M- system; it is sometimes even called the auditory transient or magnocellular system (Rauschecker, 2018; Meng and Schneider, 2022). Large neurones in the auditory brainstem (cochlear nuclei) and thalamus (the medial geniculate nucleus—MGN) are specialized for the much more precise timing required by audition than for vision. Importantly, like those in the visual LGN, they bind the M- cell specific signature antibody, CAT 301 (Hendry et al., 1988); hence histologically the auditory transient neurones can be considered part of the same neuronal lineage.

This paper is about the visual basis of dyslexia, so we will not consider the auditory analog of the visual transient system in any detail. Suffice it to be said that development of the auditory transient cells is also impaired in many dyslexics, so that anatomical (Galaburda et al., 1994), psychophysical (Hämäläinen et al., 2012) and electrophysiological (Schulte-Körne et al., 1998) studies have all found that dyslexics’ auditory temporal processing systems are abnormal. Their responses to frequency or amplitude modulated sounds are reduced compared with good readers, and the degree of this reduction predicts the impairment of their reading (McAnally and Stein, 1996; Goswami, 2014). In cohort studies these abnormalities have been shown to be evident in those at family risk of dyslexia long before learning to read begins, even in new born babies, and they strongly predict the babies’ eventual chances of suffering reading problems (Leppänen et al., 2010). Thus, in many ways, auditory temporal processing impairments parallel those seen for the visual M- system in dyslexia, and suggest that both can contribute to causing dyslexia.

Importantly, and probably as a result of their common genetic basis, these visual and auditory temporal processing deficiencies correlate strongly with each other in individuals tested for both (Talcott et al., 2002). Thus the majority of dyslexics appear to have both visual and auditory timing problems and their genetic basis probably represents the basic cause of their visual, phonological and reading difficulties.

## Magnocellular timing systems

If we define transient/magnocellular lineages by their expression of the same surface antigens, such as CAT 301 (Hockfield and McKay, 1983), they are not even confined to the visual and auditory systems. They form interconnecting networks, specialized for temporal processing, hence for sequencing, all over the brain. They track changes in light, sound, limb position, etc., for the direction of attention and the control of movement. They are found not only in the visual and auditory systems, but also in the touch and proprioceptive systems (Stoodley et al., 2000), in motor systems, cerebral cortex, hippocampus, cerebellum and brainstem (Hockfield and McKay, 1983). Their large, rapidly conducting, rapidly transmitting, neurons mediate speedy temporal processing. Everywhere, they are also extremely vulnerable. Altered magnocellular development has been reported in fetal prematurity, in fetal alcohol syndrome, DD, dyspraxia, dysphasia, ADHD, ASD, Williams syndrome, and even in schizophrenia and bipolar depression (Whitford et al., 2017).

## Docosahexaenoic acid

The high dynamic sensitivity of M- cells is mediated by their membrane ionic channels being able to open and close very rapidly. This requires the membrane to be highly flexible; and this flexibility is crucially dependent on one very important component of the diet, the omega 3 long chain fatty acid, docosahexaenoic acid (DHA) (Haag, 2003; Muskiet et al., 2004). This is normally provided by consuming oily fish. Green vegetables, flax or rape seed and seaweed all contain the shorter chain omega 3, alpha linolenic acid, but humans do not convert this into DHA very efficiently.

Unfortunately consumption of oily fish has decreased greatly over the last 50 years, particularly in disadvantaged households (Maguire and Monsivais, 2015), and so we wondered whether this might be affecting M- cell responses in the children. We therefore carried out a randomized control trial to see whether giving disadvantaged children dietary supplements of DHA could improve their M- cell responses and thereby help them to improve their reading (Richardson and Montgomery, 2005). After 3 months of consuming the supplements, the children’s single word reading improved by 9.5 months, whereas those receiving the placebo improved by only 3.3 months—a highly significant difference. Even though not all these children were classified as dyslexic, this effect of improving diet is most likely to be due to the effect of DHA improving the function of magnocellular timing neurons.

## Dyslexia strengths

Although many people are convinced that people with dyslexia often have great talents (West, 2009), most studies about it concentrate on their reading problems. But there is now evidence that, alongside their magnocellular weaknesses, people with dyslexia may demonstrate superior parvocellular performance e.g., higher sensitivity than ordinary readers to high spatial but low temporal frequency modulated gratings (Lovegrove et al., 1982), better red and green color discrimination, particularly in the peripheral visual field (Dautrich, 1993). They are also faster and more accurate at identifying “impossible figures”- drawings of objects which cannot exist in reality, such as Escher’s stairs and waterfalls (von Károlyi et al., 2003). Male dyslexics have also been shown to be particularly good at identifying shapes in ambiguous figures, remembering and reproducing designs and complex figures and at recreating and navigating in a virtual environment (Brunswick et al., 2010).

90% of the neurones that are born during early brain development fail to survive because they are eliminated in the ruthless competition to make useful connections. Hence the P- cell superiorities seen in dyslexics are not particularly surprising, since a reduced number of M-cells would leave room for more P-cells to survive and make successful connections. Probably therefore, their P- cell connectome is significantly more prolific than that of their ordinary reading peers. This outcome probably explains the very different cognitive style that characterizes dyslexic people. This style is often described as “being able to see the whole picture at once”—a “holistic” approach. Their unusual way of thinking would also account for why so many dyslexics are so remarkably successful in the arts (Wolff and Lundberg, 2002), at practical engineering (West, 1992) and in business (Logan, 2008). If they can survive their schooling which so often fails to recognize their talents, they can often achieve remarkable feats (Brock and Eide, 2012).

## Conclusion

The evidence presented here strongly suggests that DD results from impaired development of the magnocellular timing connectome *in utero* and early childhood, particularly for vision and hearing. These cortical systems are responsible for sequencing letters and word sounds for learning to read. The large variety of genetic, histological, electrophysiological, imaging, psychophysical, dietary and achievement data reviewed here, all suggests that if these visual and auditory timing/transient systems fail to develop properly or are damaged, reading becomes difficult or impossible. Precise timing of when vision alights on each letter and the order in which the sounds of a spoken word are heard, is so crucial for reading that proper development of the reading networks is permanently compromised. This coherent body of evidence establishes the neural basis of reading, and strongly suggests, contrary to much current belief, that visual and auditory timing deficits are the main causes of the phonological deficit in people with dyslexia and their reading failure.

## Author contributions

The author confirms being the sole contributor of this work and has approved it for publication.
